# Microplastic polymer type impacts water infiltration and its own transport in soil

**DOI:** 10.1016/j.isci.2025.113193

**Published:** 2025-07-24

**Authors:** Qihang Li, Anna Bogush, Marco Van De Wiel, Pan Wu, Ran Holtzman

**Affiliations:** 1 College of Resources and Environmental Engineering, Guizhou University, Guiyang 550025, China; 2 Fluid and Complex Systems Research Centre, Coventry University, Coventry, UK; 3 Centre for Agroecology, Water and Resilience, Coventry University, Coventry, UK; 4 College of Agriculture and Environmental Sciences, UNISA, Florida 1709, South Africa; 5 Key Laboratory of Karst Georesources and Environment (Guizhou University), Ministry of Education, Guiyang 550025, China

**Keywords:** Environmental science, Pollution, Soil hydrology, Soil science

## Abstract

Microplastics (MPs) pose a substantial threat to humans and ecosystems. How MPs move in soils is controlled by a large number of coupled parameters, including MPs and soil properties as well as hydrological and geochemical conditions. We conduct laboratory experiments where two commonly MPs types found in soils—polyethylene terephthalate (PET) and polypropylene (PP)—are leached into an idealized soil analog (glass beads). We use time-lapse imaging to analyze the water flow pathways and spectroscopy to measure the MPs transport. We find that MPs impede water infiltration into preferential pathways, with a stronger effect for the more hydrophobic PP, and that PET is more mobile than PP. We explain this by the stronger impedance of PP on water flow that carries the MPs (the driving force), as well as PP surface charge enhancing its adsorption onto soil particles, and its lower density that limits downward transport. These findings advance our understanding the mechanisms underlying MP transport in soils.

## Highlights

• Polyethylene terephthalate (PET) and polypropylene (PP) leached into a soil analog
• MP found to inhibit water flow rate, forcing it through preferential pathways (fingers)
• More pronounced effect for PP, which is more hydrophobic than PET
• PP surface charge and density further inhibit MP transport vs. PET

## Graphical abstract

**Figure:**
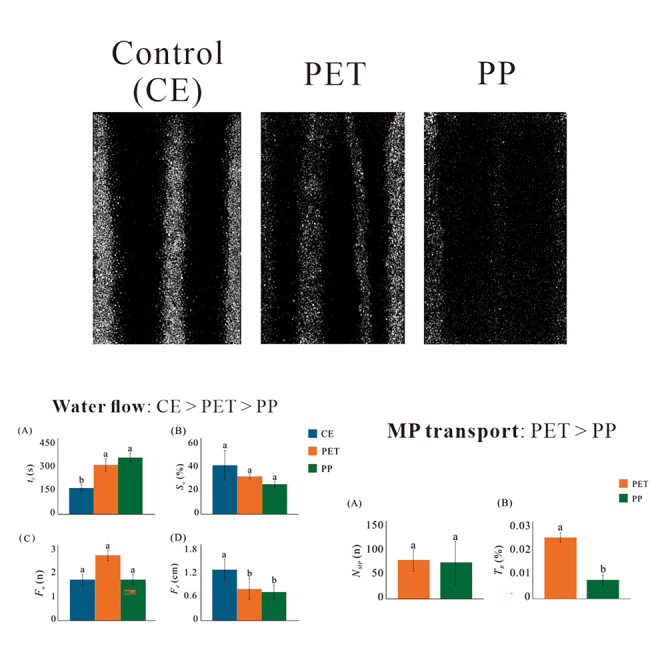

## Introduction

Plastic pollution is ubiquitous in our environment, with over 0.5 million metric tonnes of plastic waste added to the terrestrial environment every year.^1^^,^^2^ Microplastics (MPs), i.e., plastic particles of size 1–5,000 μm,^3^ negatively impact soils, e.g., inhibiting plant growth,^4^^,^^5^ mobilizing pollutants such as heavy metals and persistent organic pollutants,^6^ reducing the diversity of bacterial communities,^7^ increasing ecotoxicity, and impeding the biogeochemical cycles.^8^ The extent of these negative processes is controlled by the distribution and fate of MPs within the soil, which in turn depends on MP transport. One of the key properties affecting MPs transport is MP polymer type,^9^ which determines the MPs density and surface properties such as zeta potential, hydrophobicity, and surface roughness^10^^,^^11^^,^^12^^,^^13^ (Table 1). The MPs polymer type also impacts indirectly on soil properties, such as pH, bulk, and soil enzyme activity,^15^^,^^16^^,^^17^^,^^18^ which in turn further affect MP transport.

**Table 1:** Relative transport of different MP polymer type

Relative transport	Metric used^a^	Reasoning	Reference
PE > PP	depth reached	density	O’Conner et al., 2019^10^
PE > PET > PP	depth reached	zeta potential, hydrophobicity	Gao et al., 2021^14^; Ranjan et al., 2023^12^
PA > PE	depth reached	hydrophobicity	Gao et al., 2021^14^
PA > PET	transport rate	hydrophobicity	Cohen and Radian, 2022^13^
PVC > PLA	transport rate	zeta potential, hydrophobicity	Fei et al., 2022^11^

PE, polyethylene; PP, polypropylene; PET, polyethylene terephthalate; PA, polyamide; PVC, polyvinyl chloride; PLA, polyactic acid.

a “depth reached” and “transport rate” refer to the maximum depth in which MPs were found within the sample, and MP concentration in the effluent traversing the sample, respectively.

Transport of water and MPs have an intricate, two-way coupling. Water infiltration, the main driving force for MPs transport, strongly affects MPs fate.^9^ Qi et al.^19^ found that higher water flow rates are conducive to MPs transport. Higher water infiltration rates promote vertical MP transport, whereas lower flow rates promote horizontal MP transport.^20^ At the same time, MPs also alter water infiltration in soil, changing the direction and tortuosity of water flow by blocking pore spaces,^21^^,^^22^ and by inducing soil water repellency.^23^ MPs of smaller sizes are found to decrease water infiltration in soils.^24^

While the interdependence between water infiltration and MP mobility and the significant impact of MP type on MP mobility have been investigated, the link between these—how MP type impacts water infiltration and MP transport—remains poorly understood. Here, we address this gap by laboratory experiments using idealized soil analog, that we use to quantify transport of both water and MPs through the sample, by which we expose the links between MPs type, water flow (driving force for MPs transport) and MPs mobility, going beyond previous studies.

**Figure 1:**
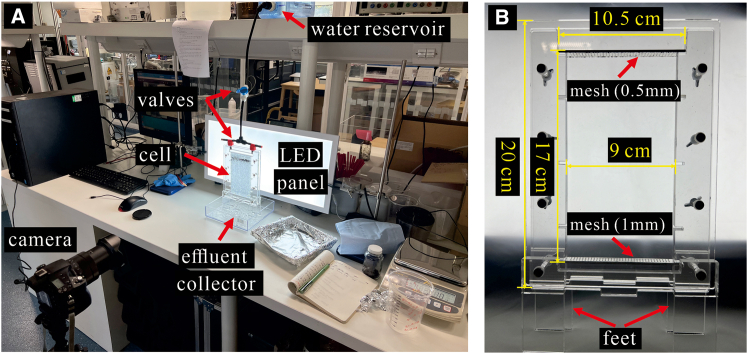
Experimental setup (A) A cell filled soil analog (glass beads) contaminated by microplastics was subjected to infiltration of a given amount of water by gravity from above. (B) Cell inner dimensions are 17 cm height by 10.5 cm width by 1 cm (out-of-plane) thickness.

We use glass beads of narrow size range as analog of e.g., sandy soil, highly purified water, and single-type MPs, packed in a quasi-2D transparent cell (Figure 1). These settings allow visual observation of water flow pathways via time-lapse imaging, while excluding interactions with various physical, chemical, and biological processes such as wide particle size distribution, intricate heterogenous mineralogy, the presence of various organic and inorganic substances, and microbial activity, all of which are unfeasible in standard column experiments using natural soils. Consequently, our approach enhances (1) our ability to isolate the *main physical* effects of the MP type on water and MP transport in the experiment and (2) repeatability of the experiments, which are otherwise highly dependent on the specific soil sample details.

## Results

### Impact of MP type on water flow

**Figure 2:**
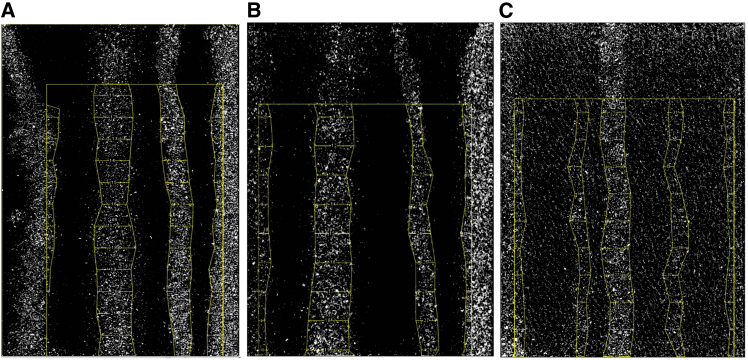
Experimental images of the water patterns We compare samples containing (A) no microplastic, (B) PET, and (C) PP. White and black pixels are the wet and dry areas, respectively. The outermost yellow rectangle is the region of interest included in the analysis (area A). Wet finger boundaries are delineated by yellow trapezoids (height increments of 0.1 cm).

**Figure 3:**
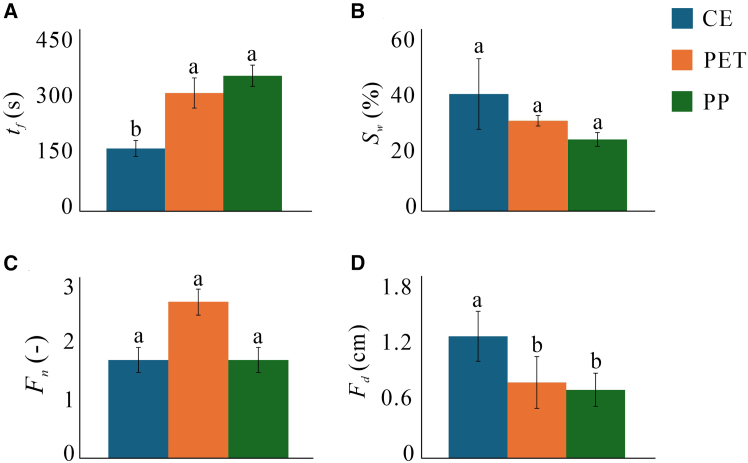
Impedance of water flow by the presence of microplastics Flow impedance shown in terms of (A) flow time (traversing the sample), tf; (B) water saturation, Sw; (C) number of fingers, Fn; and (D) finger width, Fd. Error bars indicate standard deviations across 3 repeated experiments under identical conditions. Different letters (a, b) indicate a statistical significance difference (*p* < 0.05).

Qualitative evidence of the impact of MPs addition to the sample is the alteration of water flow pathways (Figure 2; data in Table S2 in SM). To quantify the impact of MPs on the water flow, we use (1) the time it takes for water to drain, (2) water saturation, and the (3) number and (4) width of preferential flow conduits (fingers). The drainage time *t*_*f*_ is an indication of the resistance to flow. The addition of MPs was found to increase flow resistance, with *t*_*f*_ increasing from 170 s in the control (CE) to 320 and 367 s with the addition of polyethylene terephthalate (PET) and polypropylene (PP), respectively (Figure 3A). Water saturation was found to decrease with the addition of the MPs, from 42.3% in the control to 32.6%, and 25.9% for PET and PP, respectively (Figures 2 and 3B; noting however that statistically they are not significantly different from the control [*p* > 0.05]). The addition of MPs was found to enhance preferential pathways for water flow mostly via decreasing the pathways (fingers) *width F*_*d*_ rather than their number *F*_*n*_ (Figures 3C and 3D), where *F*_*d*_ decreased from 1.31 cm (control) to 0.84 and 0.78 cm with the addition of PET and PP, respectively.

### Impact of MP type on MP transport rate

**Figure 4:**
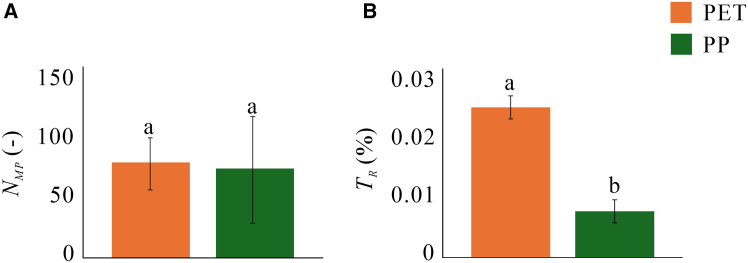
Impact of microplastics type on their transport Impact is quantified via (A) number of MPs traversing the sample, NMP; and (B) transport rate, TR. Error bars indicate standard deviations across three experiments repeated using identical conditions. The different letters (a, b) indicate a statistical significance difference (*p* < 0.05).

First, to ensure there was no arbitrary contamination in the samples (water or glass beads), we analyzed the effluent water from the control experiments (CEs). The Fourier transform infrared (FTIR) data confirmed there was no MP contamination. The MP particle counts in the effluent from the three replicates using PET (denoted PET1, PET2, and PET3) are 109, 62, and 64, vs. 133, 27, and 60 from PP1, PP2, and PP3, respectively (Figure 4A; data in Table S3 in SM). The average particle lengths of PET and PP in the effluent were 160 and 153 μm, and the average widths were 45 and 47 μm, i.e., average length-to-width ratios of 3.52 and 3.38, respectively. The volume of PET and PP were 5.21·10^7^ μm^3^ and 2.37·10^7^ μm^3^ (Table S1 in SM). In terms of mobility, PET is an order of magnitude more mobile than PP, with MP transport rates of 0.022, 0.027, and 0.024% for the PET experiments vs. 0.004, 0.009, and 0.007% for PP (Figure 4B; see also Table S1 in SM).

## Discussion

### Water flow: Impact of MP polymer type

The addition of MPs (both types) was found to enhance preferential pathways for water flow, manifested by a reduction in finger width. Water flow is proportional to the driving force (held uniform across our experiments), and permeability which is directly proportional to the amount of volume accessible to flow.^25^ Therefore, reduction in finger width while keeping the same number of fingers impedes water flow.

A similar reduction in water flow was found for the addition of polyethylene (PE) in glass beads packing.^23^ The authors explained this by trapping the hydrophobic MP within the air-water interfaces, which enhanced entrapment of air within the porous sample, hence reducing water saturation and increasing the tortuosity of flow paths. Our finding of the slower water flows in PP-contaminated samples vs. PET could be explained by the higher hydrophobicity of PP compared to PET.^14^ We note a contrasting observation of enhanced infiltration in PE-contaminated silt loam and loamy sandy soil samples, which was explained by an increase in pore sizes due to the addition of MPs, hence increasing the soil hydraulic conductivity.^26^ This difference could be due to aggregates forming between fine soil particles and MPs in natural silt loam and loamy sand soils used in a study by Xing et al.^26^

The effect of MPs in terms of increasing the degree of irregularity of the infiltration front (i.e., its roughness) and enhancing preferential pathways observed in our experiments, agrees with previous experiments.^24^^,^^27^ While Fang et al.^24^ explained this by a higher proportion of hydrophobic surfaces in aggregates containing the (hydrophobic) MPs, Guo et al.^27^ attributed this to MPs filling pre-existing macropores when they are in the soil, which hinders water infiltration. As noted in a recent review, interference among different properties (MPs, soils, and water) and mechanisms (physical, chemical, and biological) could have a substantial, non-trivial impact (denoted “co-effects” in Li et al.^9^). As an example of such co-effect, Liu et al.^28^ found that the impact of MPs on water infiltration depends on MP concentration: decreasing infiltration at small PE MP concentrations (<0.2%) due to clogging by the small MPs, vs. enhancement of infiltration for larger concentrations where MPs aggregate with soil particles, forming large macropores between the aggregates. In the current study, with MP concentrations of 1%, we did not observe such behavior, because the glass beads cannot form large macropores and aggregates with MPs.

### MP mobility: Impact of MP type

In our experiments, PET MP transport was higher than PP. This can be due to their bulk and surface properties, in terms of density as well as surface charge and roughness. The positively charged PP particles are readily adsorbed into glass beads, reducing their mobility. In contrast, as glass beads and PET both carry a negative surface charge, they repel each other. Also, the surface roughness of PP MPs is higher than PET MPs, which makes PP more easily interlocked with the glass beads, impeding their transport.^12^ Our findings agree with the experiments in Gao et al.^14^ and Ranjan et al.,^12^ which found higher mobility (in terms of maximum depth reached by the MP) of PET vs. PP, which they explained by the PET lower hydrophobicity,^14^ higher zeta potential, and lower roughness.^12^ MP transport is influenced by multiple factors, including zeta potential, shape, size, density, and surface roughness,^9^ and surface roughness is not always the dominant factor.^29^ Although direct measurements of surface roughness were not performed in this study, previous results from Ranjan et al.^12^ confirm that, within the same size range, PP MPs generally exhibit a rougher surface than PET. Additionally, the volume of PET particles in the effluent was larger than that of PP, supporting the observed greater mobility. Finally, PP is much lighter than PET, increasing the buoyancy force that impedes their downward flow.^10^^,^^30^

Our experiments suggest that, beyond the aforementioned mechanisms, MPs can also affect their transport *indirectly*, by affecting the *water flow*—the driving force for MP transport. MPs, being hydrophobic particles, induce soil water repellency and thus increase the tortuosity of the flow path.^23^ Increased tortuosity can then lead to impeded MP advancement. PP, which is more hydrophobic than PET, will thus reduce the driving force for its transport, providing an additional explanation to its observed lower mobility.

We observed high variability across replicates in both PET and PP treatments (mostly between no. 1 and nos. 2 and 3), as well as lack of clear correlation between the number of MPs in the effluent and MPs transport rate. One potential explanation for this is variability in both MPs sizes and shapes, which we could not control and monitor. In preparing the MP, we sieved the particles to bound the size range to 180–250 μm; however, our FTIR analysis of the effluent showed the existence of several smaller MPs, which may have been adsorbed to the surface of the large particles. Such smaller particles are expected to have a greater impact on MPs number than on transport rate. Another plausible explanation for this variability is differences in the length-width ratio of MP particles: some MP particles found in the effluent from PET2, PET3, PP2, and PP3 samples are elongated (i.e., with a large length-width ratio; cf. Table S1). Such elongated shape can improve the particle transport, by allowing them to align with the flow direction and pass through narrow pores.^31^ Here, the smaller width of the PET MPs in our experiments can be the reason for their higher transport rate vs. PP (Table S3).

### Summary and conclusions

We examined experimentally how different MP polymer types affect the flow of water and consequent transport of the MPs. We quantified the water saturation, the uniformity of the flow (in terms of finger indicating preferential pathways), the time of flow, and the rate of MP transport through thin transparent cell-packed glass beads. We found that MPs inhibit water flow by making it more preferential, with the more hydrophobic PP showing a more substantial effect than PET. PP exhibited a lower MP transport rate, which could be due to a combination of (1) its impeding effect on water flow—the driving force for MP transport—by increasing flow tortuosity and fingering, as well as (2) its greater tendency to adsorb onto soil’s porous matrix (due to its positive surface charge), higher surface roughness, and lower density (impeding downward transport). By quantifying these coupled processes under simplified settings, this study offers a mechanistic explanation for differences in water infiltration and MPs mobility in soils associated with MPs type.

Further research is needed to entangle the effects of MPs on water flow and on their own transport, to address a fundamental question: Do preferential pathways, induced by hydrophobic particles such as MPs themselves, impede or enhance MP transport? Further important insights can be gained by investigating how other MPs properties (e.g., size, surface roughness, and shapes) interplay with MPs type to affect water flow and MP transport, and how this is exacerbated by the strong coupling (“co-effects”^9^) among various MPs and soil properties and environmental conditions. The complexity of natural settings, including (1) soil texture, composition, and mineralogy; (2) the highly non-uniform spatiotemporal nature of soil water flow and geochemistry; and (3) the various coupled biogeochemical processes that can affect MP transport, makes understanding of MP transport in soils a daunting challenge, which requires collaborative interdisciplinary research combining modeling with laboratory experiments (using both idealized proxies as well as natural samples) and field studies. Advancing our understanding of the mechanisms underlying MPs transport in soils could enhance our ability to mitigate MP pollution; for instance, by manipulating MP properties to tune water infiltration and subsequent MP migration and fate.^32^

### Limitations of the study

This study used idealized system for the soil (clean glass beads of narrow size range), water (highly purified), and single-type MPs. While this is a useful and necessary first step towards understanding MP transport in soils, natural systems are appreciably more complex in terms of soil properties (e.g., wide range of pore sizes, mineralogy, and organic and inorganic constituents) and soil solution geochemistry. Additionally, the methodology for preparation of MPs used here could not provide a full control and knowledge of MPs sizes and shapes. As variability in these properties can influence MPs transport behavior, it limits our ability to isolate the effects of specific MPs characteristics.

## Resource availability

### Lead contact

Requests for further information and resources should be directed to and will be fulfilled by the lead contact, Ran Holtzman (ran.holtzman@coventry.ac.uk; rholtzman4@gmail.com).

### Materials availability

This study did not generate new unique reagents.

### Data and code availability

• All raw and processed data reported in this paper will be shared by the lead contact upon request.
• This study did not generate an original code.
• Any additional information required to re-analyze the data reported in this paper is available from the lead contact upon request.

## Acknowledgments

Q.L. and R.H. acknowledge support from the China Scholarship Council-Coventry University joint research scholarship (CSC no. 202206670004). R.H. also acknowledges support from the 10.13039/501100000266Engineering and Physical Sciences Research Council (EP/V050613/1), and from the Royal Society (IES\R2\232054). We thank Ali Saeibehrouzi (Coventry University) for performing a zeta potential analysis.

## Author contributions

Q.L., conceptualization, methodology, investigation, formal analysis, validation, visualization, writing – original draft preparation, and writing – review & editing; A.B., conceptualization, methodology, investigation, formal analysis, validation, resources, writing – review & editing, and supervision; M.V.D.W., conceptualization, methodology, investigation, formal analysis, validation, writing – review & editing, and supervision; P.W., supervision; R.H., funding acquisition, project administration, conceptualization, methodology, investigation, formal analysis, validation, resources, writing – review & editing, and supervision.

## Declaration of interests

The authors declare no competing interests.

## STAR★Methods

### Key resources table

**Table:** 

REAGENT or RESOURCE	SOURCE	IDENTIFIER
**Software and algorithms**		

ImageJ software	https://release-monitoring.org/project/98365/	RRID:SCR_003070
Imaging Edge software	https://imagingedge.sony.net/en/ie-desktop.html	Version 3.8.01 (released June 2025)
OMNIC™ Picta™ Software (Thermo Scientific, USA)	https://assets.thermofisher.com/TFS-Assets/MSD/Specification-Sheets/nicolet-in10-mx-infrared-imaging-microscope-ps51511.pdf	Version 1.9 (released December 2023)

### Method details

#### Materials

Polyethylene Terephthalate (PET) and Polypropylene (PP) were used in this study because they are the common plastic polymers found in soils.^33^ PET and PP were sourced from water bottles and plastic welding rods, respectively. To confirm the polymer types of both selected materials, a piece of both materials was analyzed using the Nicolet iN10 MX Fourier Transform Infrared Spectroscopy (FTIR) Imaging Microscope (Thermo Scientific, USA) in Attenuated Total Reflectance (ATR) mode. The spectrum of both materials is shown in Figure S1 in Supplementary Materials (SM). To obtain a desired MPs size range, the plastic source material was cut and ground into small fragments (using Wahl ZX889 grinder), which were sieved to retain 180-250 μm particles—a common size range of MPs in soils.^4^^,^^34^ The densities of PET and PP are 1.4 and 0.833 g/cm^3^, respectively. The measured zeta potentials for PET and PP are −0.51 and 6.53 mV, respectively (at pH 7 and zero salinity; see Table S4 in SM).

Glass beads (Sigmund Lindner GmbH, Germany) properties are: diameter 2 mm ± 0.2 mm, density 2.5 g/cm^3^ and zeta potential −10.9 mV (at pH 7 and zero salinity; see Table S4 in SM). The beads were washed using Milli-Q water before the experiment to avoid contamination that could affect MPs transport. Similarly, to avoid effects of water impurities, the experiments were conducted using Agilent Infinity Lab HPLC (high-performance liquid chromatography) Water (Fisher Scientific UK) 0.2 μm prefiltered providing low level of impurities.

#### Experimental setup and procedure

The setup consists of a quasi-2D cell, water reservoir, effluent collection container, LED light panel, and a camera (Figure 1). The cell, made of transparent plexiglass cell walls to allow visual observation of transport, has inner dimensions of 17 cm height, 9 cm width, and 1 cm thickness (out-of-plane). The cell has a fine mesh at the top (∼0.5 mm pore diameter) and bottom (∼1 mm), to ensure water is distributed evenly from the top and that the beads are not carried out with the water at the bottom. The cell is kept above the bottom collection container by raised feet to avoid effluent re-entering the cell from below. A 80 cm × 40 cm LED light panel was fixed behind the cell to improve contrast for the identification of the different materials. A digital SLR camera (SONY RX10 with 24–200 mm f2.8 lens) was used for timelapse imaging. Time-lapse images were taken every 10 s (using Imaging Edge software).

The contaminated soil analog was prepared by first gently pouring and tapping (i) 205 ± 1 g glass beads into the bottom of the cell, forming a packing layer ∼14 cm height (porosity of ∼35%), followed by (ii) a pre-mixed layer (prepared in a separate container) consisting of 0.3 g MP and 30g of glass beads (1%, w/w, ∼2 cm in height), and (iii) another ∼10 g of glass beads (∼0.7 cm height) on top. The latter was added to prevent disturbance of the MP-contaminated layer directly by water applied from above. The cell was filled with beads almost all the way to its top, keeping a small empty gap to ensure water were retained without overflowing when injection started.

In each leaching experiment, 600 mL of water was transferred by a graduated cylinder to a reservoir above the cell. A volume of 600 mL was found (by trial and error) to (i) fully saturate the porous sample and (ii) provide enough excess for a consistent and visible drainage flow throughout the experiment. The experiment was stopped when the water added from above (600 mL) infiltrated out of the cell. We note that while this procedure allows gravity-dominated drainage to complete, it does not ensure that the much slower capillary-dominated drainage is completed, and therefore small volumes of residual moisture may be retained by capillary forces. We note however that this residual moisture does not influence the main short-term transport characteristics including flow paths and finger formation – the focus of this study.

During leaching, the valves were adjusted to ensure the water in the holding space above the top mesh did not overflow. The height of the water holding space above the top mesh was approximately ∼2 cm, consistently across all experiments. In our experimental design, we chose to maintain a fixed water level above the porous sample (the driving force for gravitational drainage), such that the resulting infiltration rate through the sample varied depending on its transport properties (controlled by MPs type). The time required for infiltration was measured as output. The effluent water contaminated with leached MPs was collected from a container underneath the cell, from which it was transferred to clean glass bottles for analysis.

### Quantification and statistical analysis

#### Repetitions to quantify variability

For each MP type, experiments were replicated three times, to quantify variability (mean and standard deviation). Additionally, triplicate control experiments (CE; without microplastics) were conducted under the same experimental conditions. All data (water flow area, MP transport rate) are presented as mean ± standard deviation (Std). One-way ANOVA was used to assess significant differences among multiple groups at a probability level (P) < 0.05.

#### Image analysis

*ImageJ* (1.53a) was used to quantify the water spatial distribution, by computing the total saturation and width of preferential pathways, as follows. First, all images were converted to grayscale. We analyze the image sequence halfway through the experiments where the flow rate became steady. To exclude boundary effects, we perform all computations on the central part of the original image, excluding (i) the saturated area at the cell bottom; (ii) the top part where MPs were seeded; (iii) area along the sides of the cell (see Figure S2 in SM).

The water saturation (*S*_*w*_) is evaluated here from the wetted area (*A*_*w*_) normalized by the total area if interest *A*, *S*_*w*_ = *A*_*w*_*/A* (see Figures 2 and S3 in SM). To obtain a quantitative description of the irregularly shaped water pathway, we dissect the images into *n* horizontal sections of height 1 cm. For each flow finger, a representative width, *D*, is determined as the average of the section widths, *D*_*i*_, where *i* is the section index (*i* = 1, 2, …, *m*, with *m* being the number of sections in which the finger persists); see Table S2 in SM. The diameter reported from every group of experiments for a given MP type, *F*_*d*_*,* is calculated as the arithmetic mean of measured average diameters in the three replicate experiments. The fingers in the cell edges were excluded to compensate for edge effects. The number of finger pathways in each group of experiments, *F*_*n*_, was calculated as the average of the number of fingers in each of the three repetition experiments.

#### Effluent collection and analysis

Each water effluent was filtered through a membrane filter with a pore size of 0.2 μm (Whatman AnoDiscTM 13, GE Healthcare Life Sciences, Germany) using a prewashed vacuum-enhanced glass filtration system. The filter was transferred to a prewashed glass petri dish to dry at room temperature. Each filter was analyzed using a Nicolet iN10 MX FTIR Imaging Microscope fitted with a liquid nitrogen-cooled MCT detector (Thermo Scientific, USA) in a transmittance mode and assisted with the Particle Wizard option in the OMNIC™ Picta™ Software (Thermo Scientific, USA) to identify the number of MP particles, their size (length and width), morphology, and polymer type. We identified plastic types by comparing the generated sample spectra with IR spectra reference libraries (Thermo Scientific, USA), excluding results with less than 70% matching confidence level (Table S1 in SM). The zeta potential of MPs and glass beads was analyzed using Zetasizer Nano ZS90 (Malvern, UK).

#### Evaluating MP transport rate

The MP transport rate, *T*_*R*_*,* was evaluated from the weight ratio of MPs in the effluent, *W*_eff_, vs. total MPs seeded initially, *W*_tot_,

(Equation 1) $$T_{R}=W_{\text{eff}}/W_{\text{tot}\;.}$$

Since it is technically difficult to weigh the minuscule mass of MPs in the effluent, we evaluate it from its volume, *V*_eff_*,* and density, $\rho_{MP}$,

(Equation 2) $$W_{\text{eff}}=V_{\text{eff}}\;\rho_{MP.}$$

The total volume of the MPs in the effluent is the sum of the volumes of each MP found in the effluent, *V*_*MP*_, computed assuming a cylindrical shape of a circular cross-section, because the action of the grinder rounds the elongated body of the MPs,

(Equation 3) $$V_{MP}=\pi {\left(d/2\right)}^{2}l$$

where *d* and *l* are the particle’s width and length, measured using FTIR (Table S1 in SM).

## Supplemental information

Document S1. Figures S1–S3, and Tables S2–S4

Table S1. Excel file providing the width, length, and transport rate calculation of collected MPs, related to Sections Results and Methods Details
