# Supplementary material for: Microplastic polymer type impacts water infiltration and its own transport in soil
Source: iScience. 2025 Jul 24;28(9):113193. doi: 10.1016/j.isci.2025.113193 (PMC12356328; doi:10.1016/j.isci.2025.113193)
Supplement: Document S1. Figures S1–S3, and Tables S2–S4 [file mmc1.pdf]

iScience, Volume *28*

Supplemental information

Microplastic polymer type impacts water infiltration and its own transport in soil

Qihang Li, Anna Bogush, Marco Van De Wiel, Pan Wu, and Ran Holtzman

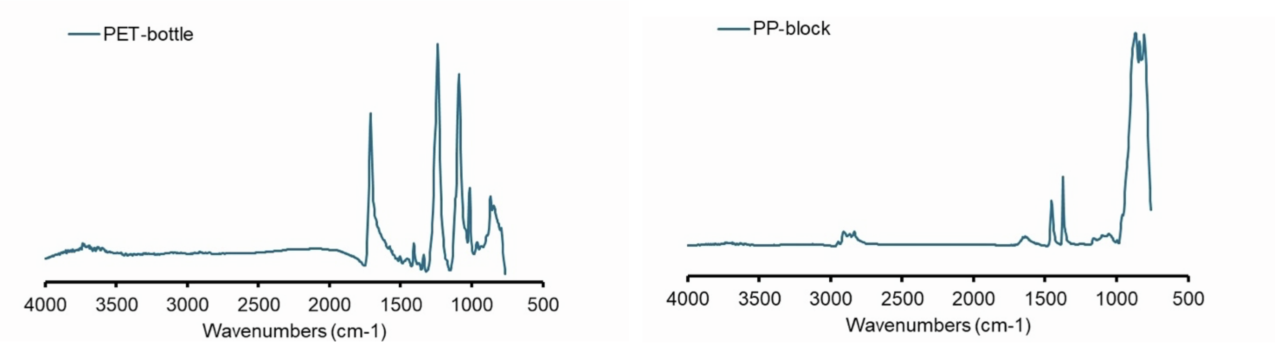

Fig. S1. FTIR analysis to determine microplastic polymer type (related to Section Methods Details/Materials). Analysis performed on a piece of the source material: PET bottle and PP block. The FTIR analysis (Nicolet iN10 MX FTIR Imaging Microscope; ThermoScientific, USA) was done using Attenuated Total Reflectance (ATR) mode.

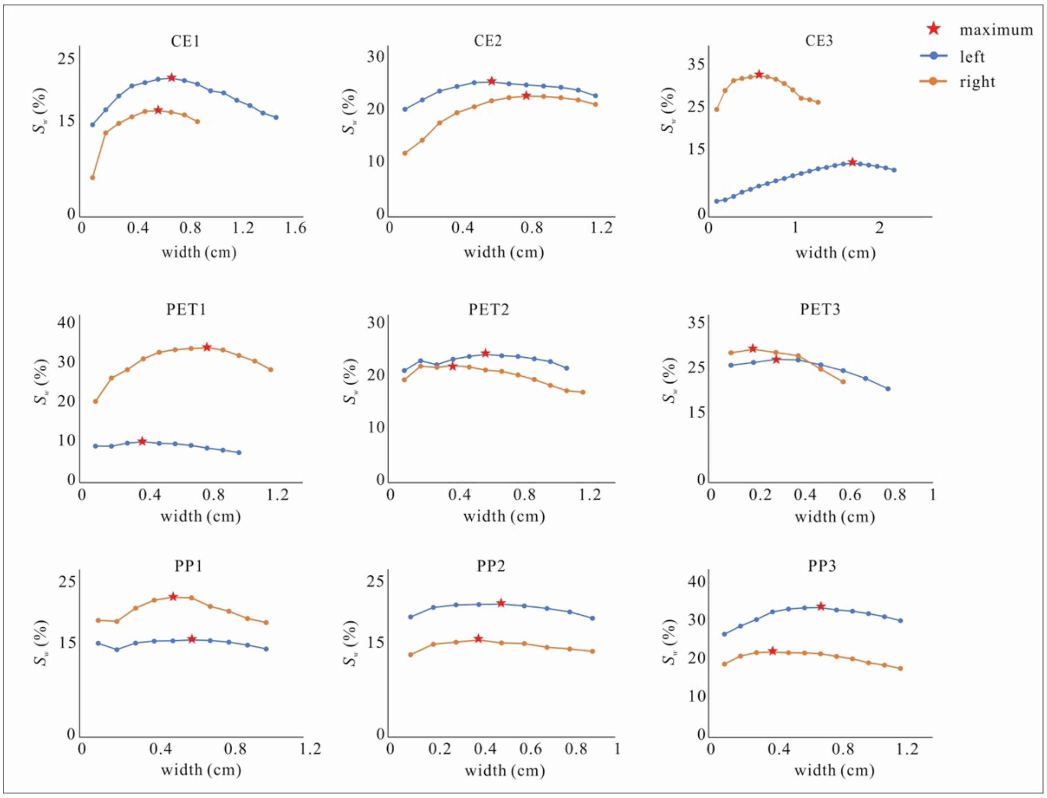

Fig. S2. Saturation profiles used to determine the region of interest (RoI; related to Section Methods Details/Image analysis). For each of the 9 experiments (PP, PET, and CE, 3 repetitions each), we determine a RoI for the quantitative analysis to minimize edge effects (of water flow close to the cell edges). We dissect the cell into columns of 0.1 cm width and compute the saturation in each column (shown here per unit thickness, i.e. the product of column width and cell height). RoI boundaries (red star) are the two columns providing the largest saturation from each side of the cell.

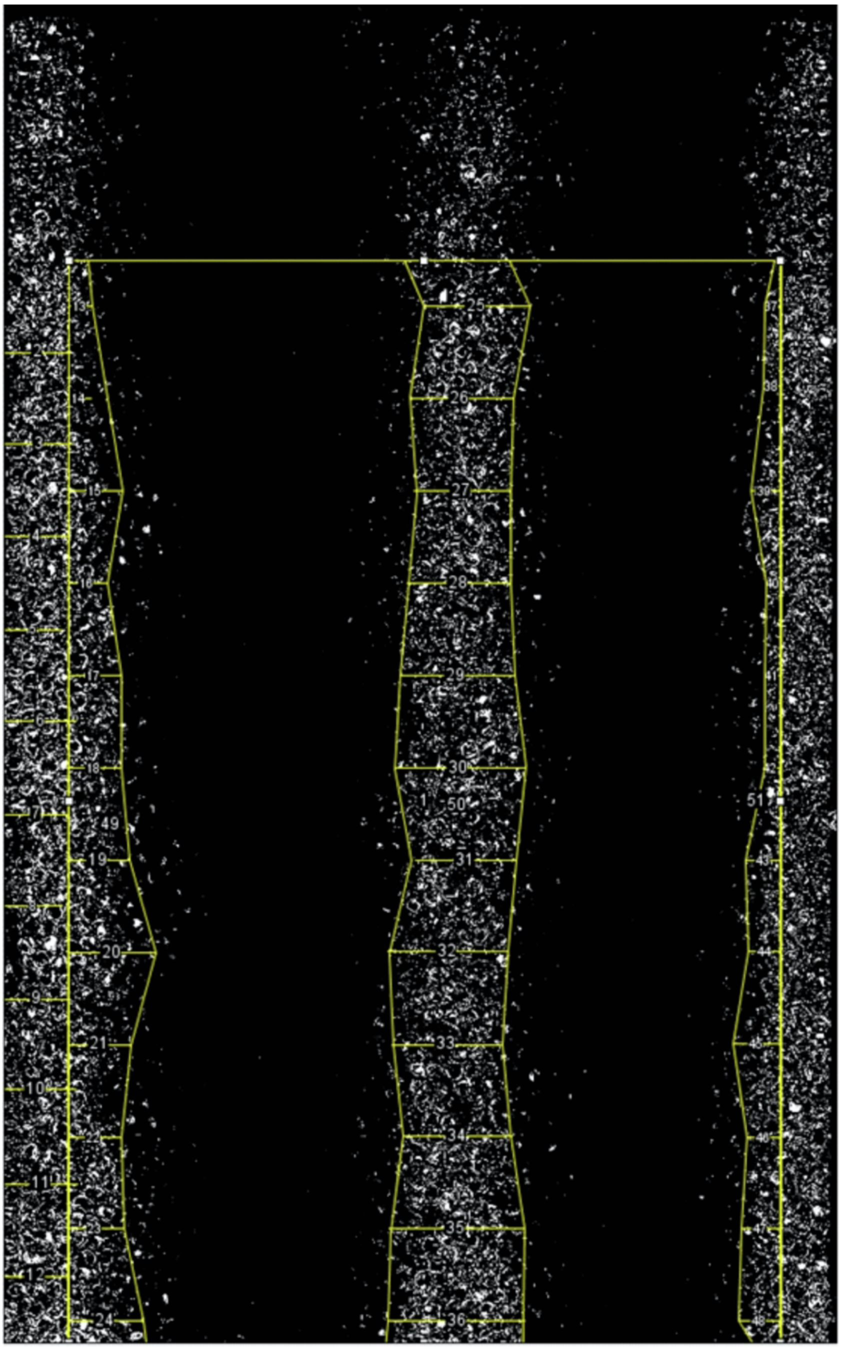

Fig. S3. Image analysis: An example interest (related to Section Methods Details/Image analysis). White and black pixels are the wet and dry areas, respectively. The outermost yellow rectangle is the region of interest analyzed (total area A in main text). Wet finger boundaries are delineated by yellow trapezoids (height increments of 0.1 cm).

Supplementary Tables

Table S2. Water flow analysis, including water saturation, S_w, flow time (traversing the sample), t_f, width of finger i, D_i, and average finger width, F_d (related to Section 2.1 “Impact of MP type on water flow”).

Experiment		S_w (%)	t_f (s)		D_i			F_d (cm)
					i=1	i=2	i=3	
CE1	27.39		150		1.23			
CE2	58.44	42.27±12.71	200	170±21.6	1.68	1.51		1.31±0.21
CE3	40.98		160		1.31	0.87		
PET1	33.64		320		1.36	0.64		
PET2	29.98	32.62±1.89	270	320±40.8	0.64	0.88	0.51	0.84±0.13
PET3	34.25		370		1.09	0.92	0.5	
PP1	24.26		400		0.74	0.75		
PP2	29.41	25.87±2.5	370	366.7±28.7	0.4	0.88		0.78±0.12
PP3	23.95		330		0.94			

Table S3. Transport rate of PET and PP, computed as weight ratio of PP traversing the sample (out of the 0.3 g added initially). Related to Section 2.2 “Impact of MP type on MP transport rate”.

Experiment	Particles (-)	Average length (μm)		Average width (μm)		Length/width (-)		Weight (g)	Transport rate (%)	
PET1	109	145		42		3.45		6.61E-05	0.022	
PET2	62	149	160±18	47	45±2	3.17	3.52±0.32	8.21E-05	0.027	0.024±0.002
PET3	64	185		47		3.94		7.06E-05	0.024	
PP1	133	113		29		3.90		1.22E-05	0.004	
PP2	27	199	153±35	65	47±15	3.06	3.38±0.37	2.68E-05	0.009	0.007±0.002
PP3	60	147		46		3.20		2.02E-05	0.007	

Table S4. Zeta potential (in mV) of glass beads, PET, and PP (at pH = 7 and zero salinity). Related to Section Methods Details/ Materials.

	Glass beads	PET	PP*
experiment #1	-13	-0.47	8
experiment #2	-7.6	-0.5	4.9
experiment #3	-12	-0.56	6.7
Average	-11	-0.51	6.5
STD	2.9	0.043	1.52

* While PP typically has a negative zeta potential under neutral pH and low ionic strength conditions, positive values has been measured before (e.g. Li et al., 2022, *Environment international*, 161:107146; DOI 10.1016/j.envint.2022.107146). It is known that chemical heterogeneities on PP surface could reverse its surface charge (Jiang et al., 2021, *Water Research*, 196:117016; DOI: 10.1016/j.watres.2021.117016). In our study, spectral analysis (Fig. S1) indicates that the PP material was not chemically pure, suggesting the presence of additives or surface modifications, which could explain the measured positive zeta potential.
